# Editorial: Synergistic approaches to managing Gram-negative bacterial resistance

**DOI:** 10.3389/fcimb.2025.1645251

**Published:** 2025-07-24

**Authors:** Andreas Erich Zautner, Hani Harb, Boyke Bunk, Melanie Lynn Conrad, Percy Schröttner

**Affiliations:** 1 Institute of Medical Microbiology and Hospital Hygiene, Medical Faculty, Otto von Guericke University Magdeburg, Magdeburg, Germany; 2 Institute for Medical Microbiology and Virology, Faculty of Medicine and University Hospital Carl Gustav Carus, Technische Universität Dresden, Dresden, Germany; 3 Bioinformatics, Leibniz Institute DSMZ-German Collection of Microorganisms and Cell Cultures GmbH, Braunschweig, Germany; 4 Neonatology and Pediatric Intensive Care, Faculty of Medicine, University of Augsburg, Augsburg, Germany; 5 Institute of Microbiology, Infectious Diseases and Immunology, Charité-Universitätsmedizin Berlin, Corporate Member of Freie Universität Berlin, Humboldt-Universität zu Berlin, Berlin Institute of Health, Berlin, Germany; 6 Institute for Clinical Chemistry and Laboratory Medicine, Faculty of Medicine and University Hospital Carl Gustav Carus, Technische Universität Dresden, Dresden, Germany

**Keywords:** Gram-negative bacteria, antimicrobial resistance, genomic studies, phenotypic studies, virulence, pathogen-host interaction

Multiresistant Gram-negative bacterial pathogens pose a major threat to global health (Huttner et al., 2013; Macesic et al., 2025), and the continuous increase in antimicrobial resistance, coupled with the very limited introduction of new antibiotics, exacerbates this situation (Aslam et al., 2018). One effective way to counteract antimicrobial resistance is through containment measures that can be implemented at local, regional and international levels. However, such measures require the collection of epidemiological data to deduce appropriate strategies, which can be very time consuming. In addition to the development of new antimicrobial drugs, ongoing evaluation of further treatment strategies is crucial. Notably, phage therapy has re-gained importance in recent decades (Slopek et al., 1983), however, to successfully implement such alternatives, detailed knowledge of bacterial pathogenicity is essential. This includes genomic data, as well as knowledge regarding how pathogenic species interact with the host microbiome and immune system.

This Research Topic highlights current research in this field and emphasizes the threat of antibiotic resistance to public health. A major focus of this Research Topic was placed on *Klebsiella pneumoniae*. In this context, epidemiological data were collected on the distribution of various resistance genes, and fundamental research contributing to a better understanding of pathogenicity was presented (Li et al., Li et al., Zhong et al.). Of particular note, regarding the genetics of hypervirulent *K. pneumoniae*, Yan et al. demonstrated the importance of the *iuc*A gene for the expression of the hypervirulent pathotype. Additionally, Klaper et al. proposed the existence of three *K. pneumoniae* pathovars (classical *K. pneumoniae*, ESBL-positive, and hypervirulent *K. pneumoniae*). Their research revealed that hypervirulent strains evade phagocytosis by macrophages and exhibit cytotoxic potential.

Further studies in this Research Topic investigated risk factors for infection with *Acinetobacter baumanii* in pediatric patients, as well as patients critically ill with COVID-19 (Wang et al., Ghamari et al.). An epidemiological study reported on patients in Lithuania who experienced invasive infection caused by *Neisseria meningitidis* (Ghamari et al.). Moreover, a new resistance mechanism to fosfomycin in *Morganella morganii* was identified and a new human pathogenic species of the genus *Stenotrophomoas* was introduced into the taxonomy (Zhang et al., Li et al.). The findings presented in this Research Topic highlight critical areas for research, hospital hygiene and public health initiatives, indicating which isolates and pathovars should become a primary focus in the future.
